# Paternal postnatal depression and child development at age 7 years in a UK-birth cohort: the mediating roles of paternal parenting confidence, warmth, and conflict

**DOI:** 10.3389/frcha.2025.1650799

**Published:** 2025-09-12

**Authors:** Iryna Culpin, Rebecca M. Pearson, Nicky Wright, Alan Stein, Marc H. Bornstein, Henning Tiemeier, Eivor Fredriksen, Jonathan Evans, Tina Miller, Esther Dermott, Jon Heron, Hannah M. Sallis, Gemma Hammerton

**Affiliations:** ^1^ Department of Child and Family Health, Florence Nightingale Faculty of Nursing, Midwifery and Palliative Care, King’s College London, London, United Kingdom; ^2^ Centre for Academic Mental Health, Population Health Sciences, Bristol Medical School, University of Bristol, Bristol, United Kingdom; ^3^ Department of Psychology, Manchester Metropolitan University, Manchester, United Kingdom; ^4^ Medical Research Council Integrative Epidemiology Unit at the University of Bristol, Population Health Sciences, Bristol Medical School, University of Bristol, Bristol, United Kingdom; ^5^ Department of Psychology, University of Leeds, Leeds, United Kingdom; ^6^ Department of Psychiatry, University of Oxford, Oxford, United Kingdom; ^7^ MRC/Wits Rural Public Health and Health Transitions Research Unit (Agincourt), School of Public Health, Faculty of Health Sciences, University of the Witwatersrand, Johannesburg, South Africa; ^8^ Eunice Kennedy Shriver National Institute of Child Health and Human Development, Bethesda, MD, United States; ^9^ Institute for Fiscal Studies, London, United Kingdom; ^10^ ECD Parenting Programmes, UNICEF, New York City, NY, United States; ^11^ Department of Child and Adolescent Psychiatry, Erasmus University Medical Center, Rotterdam, Netherlands; ^12^ Department of Social and Behavioral Science, Harvard T.H. Chan School of Public Health, Boston, MA, United States; ^13^ Department of Psychology, University of Oslo, Oslo, Norway; ^14^ School of Social Sciences, Oxford Brookes University, Oxford, United Kingdom; ^15^ School for Policy Studies, University of Bristol, Bristol, United Kingdom

**Keywords:** ALSPAC, population-based study, paternal postnatal depression, child development, father-child conflict and warmth, paternal parenting confidence

## Abstract

**Introduction:**

Paternal postnatal depression (PND) and its likely adverse impact on child development are receiving increased attention. However, research that examines processes transmitting risks of paternal PND to adverse child outcomes remains limited.

**Methods:**

This study examines pathways from paternal PND (Edinburgh Postnatal Depression Scale; 8 months) to child emotional and behavioral development (Strengths and Difficulties Questionnaire; 7 years) through paternal parenting confidence, warmth, and father-child conflict (birth-4 years) in a UK-based birth cohort, the Avon Longitudinal Study of Parents and Children (*N* = 9,628). Analyses were adjusted for socioeconomic, familial, parental, and child characteristics, including maternal PND during early postnatal period.

**Results:**

Adjusted models revealed evidence of total associations between paternal PND, child emotional symptoms, peer problems, and hyperactivity (albeit with wide 95% CIs), but not conduct problems. Indirect effects emerged from paternal PND to child emotional symptoms, hyperactivity, and peer problems through the combination of all paternal parenting factors, with no evidence of direct effects. Specificity analyses revealed indirect effects through paternal parenting confidence and father-child conflict in the associations between paternal PND and child emotional symptoms, hyperactivity, and peer problems (albeit with wide 95% CIs).

**Conclusions:**

Targeted intervention to increase paternal parenting confidence and decrease father-child conflict may improve outcomes in children whose fathers experience postnatal depression.

## Introduction

Growing evidence suggests that fathers are at increased risk of depression during their partner's pregnancy and the postnatal period (1). Meta-analyses have estimated the prevalence of paternal depression to be 9.76% during pregnancy and 8.75% during the postnatal period (2), peaking during the third trimester (9%–12%; 3) and again around 3 to 6 months postnatally (up to 26%; 4). The prevalence estimates of paternal depression in pregnancy and the postnatal period are higher than in the general adult population (3, 5), with the transition to parenthood and maternal postnatal depression (PND) increasing the risk of paternal postnatal depression (PND; 3, 4, 6). These prevalence estimates indicate that paternal depression in pregnancy and the postnatal period represent a substantial public health concern, warranting close and separate examination for their potential consequences for child development.

Increasingly, studies report consistent associations between paternal PND and increased risk of adverse child development (7), including emotional and behavioral difficulties (8, 9), peer problems (10), and hyperactivity (11). However, few studies examine processes transmitting risks from paternal PND to adverse child outcomes (7). Meta-analyses highlight genetic contributions in the inter-generational transmission of depression, with twin studies estimating its heritability at approximately 30%–40% (12). Nonetheless, depression is a complex polygenic trait influenced by many small effect genetic variants (13). Environmental mechanisms also play a role in risk transmission, including those in the immediate family environment (14, 15).

For instance, negative influences of paternal PND on child development may be transmitted through adverse effects on parenting behaviors and involvement (8, 16), including higher levels of father-child conflict, criticism, and harsh discipline and lower levels of father-child enjoyment and warmth (17). In turn, lower quality and quantity of paternal involvement (e.g., paternal non-involvement) are negatively related to emotional and behavioral child development (18, 19), thus potentially mediating the risk of depression transmission from father to child. To date, limited research has examined different dimensions of paternal involvement as potential mechanisms of risk transmission in associations between paternal PND and child development, with most studies focusing on single aspects of the father-child relationship (e.g., father-child conflict; 20) or a composite measure of paternal involvement (e.g., sum-score; 19). Conceptualizing and operationalizing father involvement as a single construct or a composite measure (unidimensional approach) may represent conceptual and methodological limitations. Conceptually, definitions of paternal involvement long ago departed from the traditional breadwinning roles and the mere time fathers and children spend together (21–23). Contemporary theories of fathering conceptualize paternal involvement as a multidimensional construct, encompassing distinct cognitive and affective/behavioral dimensions (24). The cognitive dimensions include thoughts and feelings related to parenting, such as parenting confidence (22, 25), while the affective/behavioral dimensions capture quality of father-child relationship, such as enjoyment, warmth and conflict (24–28). Paternal parenting confidence (i.e., paternal perceptions of competence in the parenting role) is a key factor in greater paternal involvement (29), playing an important role in influencing child development (30, 31). Affective/behavioral dimensions, such as enjoyment, warmth, and conflict are also important determinants of child development (25, 32, 33). Methodologically, these distinct dimensions may be differentially associated with child development. Disaggregating contributions of specific paternal cognitions and practices to child development would advance our understanding of unique paternal contributions to child outcomes, while also informing the design of targeted preventative and intervention programs (24). A systematic review of qualitative studies of father involvement during early childhood suggested that the roles of these dimensions in influencing paternal parenting behaviors and child development should be explored in future research (26).

In previous analyses, we derived several child-focused and mother-influenced dimensions of paternal involvement and examined their role in the associations between maternal PND and child development (18). Child-focused paternal involvement captured behavioral (e.g., direct involvement in caregiving), affective (e.g., enjoyment, warmth, father-child conflict, and worries about the child) and cognitive (e.g., parenting confidence and beliefs regarding caregiving) dimensions directed at the child. In contrast, mother-influenced dimensions encompassed aspects of paternal involvement with the child through the lens of maternal expectations (e.g., maternal “gatekeeping”, managing employment and parenthood), mother-father relationship (e.g., paternal beliefs regarding mother-father relationship and its impact on parenting) and indirect material care through support of the mother (e.g., paternal help with household tasks and responsibilities). Out of all child-focused dimensions (six in total) of paternal involvement, only lower levels of parental parenting confidence, enjoyment, and warmth, and higher levels of conflictual father-child relationship were strongly associated with higher risk of adverse child development in the context of maternal PND. There was no evidence for associations between any mother-influenced dimensions of paternal involvement and child development (18).

Based on these findings and stimulated by the specificity principle (34), the present study sought to estimate the extent to which associations between paternal PND at 8 months and child emotional symptoms, conduct problems, hyperactivity and peer problems at 7 years are mediated by these specific cognitive (parenting confidence) and affective/behavioral (enjoyment, warmth, and conflictual father-child relationship) dimensions of paternal involvement during the first 4 years of the child's life (Figure 2 represents our mediation model). Quantifying the magnitudes of these associations is critical for the design and implementation of targeted preventative and intervention programs aimed at improving outcomes of those children whose fathers experience PND. The clinical importance of such interventions has been highlighted by meta-analyses pointing to the modifiable nature of parenting (35), including in the context of parental depression (36). The study sample comprised participants from a large UK population-based birth-cohort study, the Avon Longitudinal Study of Parents and Children (ALSPAC). The ALSPAC cohort is a unique and rich source of longitudinal data, which enabled us to conduct confirmatory analyses to (1) model several dimensions of paternal involvement during early childhood (birth-4 years) and child development in middle childhood; (2) estimate longitudinal associations between paternal PND, paternal involvement, and child development; and (3) account for a range of socioeconomic, familial, parental, and child characteristics, including maternal PND during early postnatal period.

## Methods

### Study cohort

**Figure 1:**
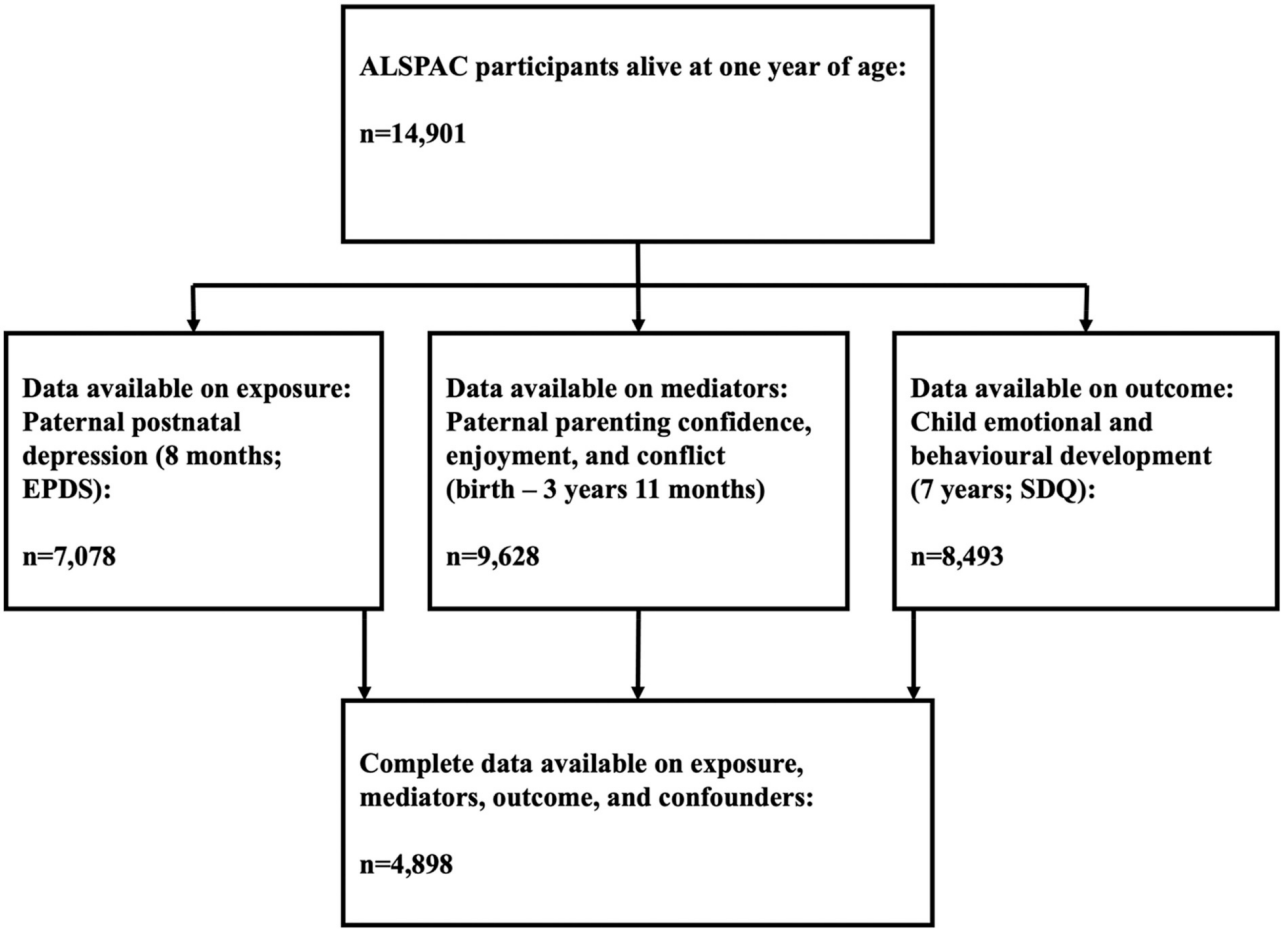
Flow chart depicting study derivation.

During Phase I enrolment, 14,541 pregnant women with expected dates of delivery between 1 April 1991 and 31 December 1992 were recruited into the ALSPAC cohort (Generation 0; G0). From the age of 7 years, children from eligible pregnancies, who were not originally enrolled, were encouraged to join the study (37). The total sample size for analyses in the ALSPAC cohort is therefore 15,447 pregnancies, of which 14,901 were alive at 1 year of age. G0 partners were invited by the mothers to complete questionnaires at the start of the study, but they were not formally enrolled in the study during Phase I enrolment. Data were collected from 12,113 partners (baseline number of partners who have had contact with the study), with 3,807 G0 partners currently enrolled in the study [37; ethnicity: White (97%); *M* age: 31 years old]. The present study sample comprised 4,898 participants with complete data on exposure, outcome, confounders, and at least one parenting item (indicators of paternal involvement) and 9,628 participants with imputed data on exposure, outcomes, and confounders (Figure 1). Demographic characteristics of the study sample are presented in the Results. Information about ALSPAC is available at https://www.bristol.ac.uk/alspac/, including a fully searchable data dictionary and variable search tool (http://www.bris.ac.uk/alspac/researchers/our-data/). Further details on the cohort profile, including G0 partners, representativeness, and phases of recruitment are described in several cohort-profile papers (37–39). Ethical approval and informed consent for the data collection were obtained from the ALSPAC Ethics and Law Committee and the Local Research Ethics Committees (http://www.bristol.ac.uk/alspac/researchers/research-ethics/). Informed consent for the use of data collected via questionnaires and clinics was obtained from participants following the recommendations of the ALSPAC Ethics and Law Committee at the time.

## Measures

### Exposure: paternal postnatal depression (PND)

Paternal depressive symptoms were measured at 8 months postnatally using the Edinburgh Postnatal Depression Scale (EPDS; 40), a 10-item self-reported questionnaire, validated and used extensively to screen for depression in men and women during the perinatal period (41). We used Confirmatory Factor Analysis (CFA) to derive a normally distributed latent trait based on the 10-EPDS ordinal response items. Similar to previous research, we dichotomized paternal depressive symptoms, derived as a continuous score, at a threshold of ≥12 (15) to examine descriptive characteristics of the study sample. Full details about the assessment of paternal depressive symptoms using the EPDS in the ALSPAC cohort, including stability across assessment points and predictive validity, are reported in Paul and Pearson (42). Throughout this manuscript, we refer to PND not as a clinical diagnosis of depression, but paternal and maternal postnatal depressive symptoms.

### Outcomes: child emotional symptoms, conduct problems, hyperactivity, and peer problems

Child development was assessed using the Strengths and Difficulties Questionnaire (SDQ; 43) completed by mothers of study children at age 7 years. The SDQ consists of 25 questions with five subscales, extensively validated to demonstrate high consistency, reliability, and diagnostic predictability among children aged 4 to 16 years (44, 45), as well as good agreement with the Child Behavioral Checklist (46). We focused on four subscales capturing emotional symptoms (5 items), conduct problems (5 items), hyperactivity (5 items), and peer problems (5 items) to derive normally distributed latent traits based on 20-SDQ ordinal response items using the CFA from those subscales. We derived sum scores of the 4 subscales to examine descriptive characteristics of the study sample.

### Mediators: paternal parenting confidence, enjoyment and warmth, and conflictual relationship with child

Potential parenting items were extracted from paternal self-report questionnaires completed by fathers on five occasions after the birth of the study child (8 weeks and 8 months postnatally, 1 year 9 months, 2 years 9 months, 3 years 11 months). We specifically focused on parenting items collected during the first 4 years of the child's life to capture possible early mechanisms of familial transmission of adverse developmental outcomes through paternal involvement and the quality of the father-child relationship. We drew on extensive empirical and sociological literature on paternal involvement in infancy (21, 24, 27, 28, 47, 48) to conceptualize the derivation of factors, including those capturing cognitive and affective/behavioral dimensions of paternal involvement directed at the child (i.e., child-focused; 18). In the first instance, extracted items were double-rated and independently assigned to theoretical dimensions by three researchers, followed by extensive discussions in a larger research group, including considerable input from experts in early child development and parenting (AS and MB). In the present analyses, we focused on three specific dimensions of paternal involvement as strong risk factors for adverse child development: paternal parenting confidence, enjoyment and warmth, and conflictual relationship with child. Derived factors, individual parenting items, age at assessment, and standardized factor loadings are presented in Supplementary Table S1.

### Baseline confounders: socioeconomic, familial, parental, and child characteristics

**Figure 2:**
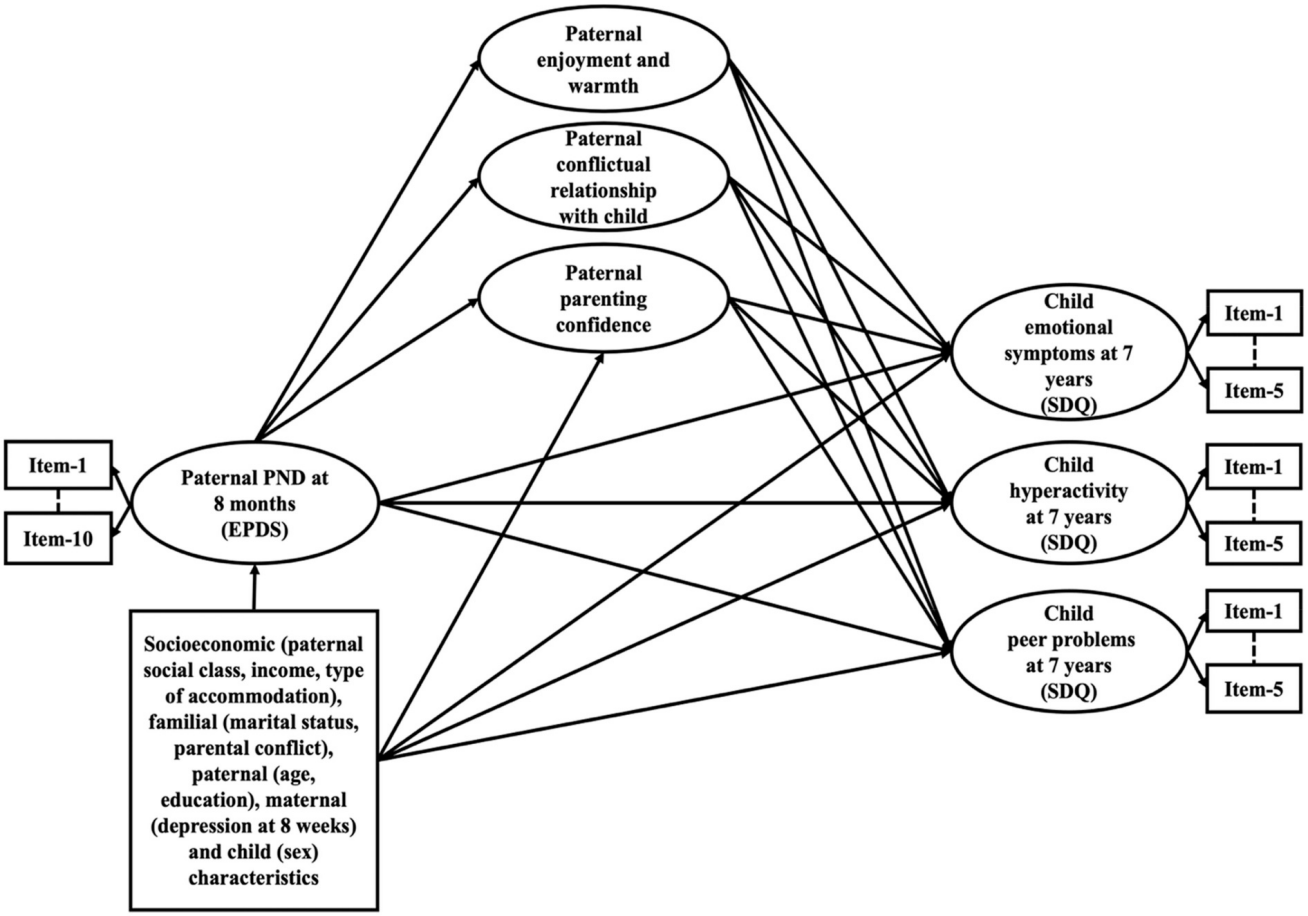
Structural equation mediation model estimating the direct effect of paternal postnatal depression (8 months) on child emotional and behavioral development (7 years), and the indirect (mediated) effects through paternal parenting confidence, enjoyment and warmth and conflictual relationship with child, adjusted for child sex, antenatal baseline confounders, and maternal PND. Note. Ovals represent latent variables. Individual items loading onto each specific factor comprising paternal involvement, error term covariances, and correlations between the factors are not shown to reduce figure complexity.

We accounted for a range of possible baseline antenatal confounders including socioeconomic, familial, parental, and child characteristics in the regression models to estimate each of the exposure-outcome, exposure-mediators, and mediator-outcome pathways (Figure 2). We adjusted our analyses for child sex to account for the importance of child characteristics in child development (15) and paternal involvement (49). Paternal age, education, disadvantaged socioeconomic background, marital status, and conflict are associated with parental PND (50), less optimal parenting practices (51), and higher risk of adverse child emotions and behaviors (52). Thus, our analyses were adjusted for a range of prospectively measured potential confounding factors extracted from self-reported paternal antenatal questionnaires, including marital status (married, never married); a continuous score capturing parental conflict (higher scores representing higher levels of conflict); paternal age in years; the highest paternal educational attainment (minimal education or none/compulsory secondary level [up to age 16 years; O-Level], non-compulsory secondary level [up to age 18 years; A-Level/university level education); paternal social class (non-manual, manual); presence of financial difficulties (no financial difficulties); and type of main dwelling/accommodation (owned/mortgaged, private/council rented).

Existing epidemiological evidence supports associations between maternal and paternal PND (53, 54), with a substantial body of evidence documenting the adverse effect of maternal PND on child development (55). Thus, our analyses also adjusted for maternal PND measured at 8 weeks postnatally using the EPDS. To make full use of variation in maternal symptoms, individual depression items were summed to derive a continuous score (range 0–30).

## Statistical analyses

### Latent factor models

Individual paternal parenting items that were theoretically relevant and had standardized loadings >0.15 were assigned to the hypothesized parenting dimensions (parenting confidence, enjoyment and warmth, conflictual father-child relationship) and modeled using CFA. We used a robust Weighted Least Square (WLSMV) estimator in M*plus* as recommended to model both categorical and continuous data (56). The CFA was also used to derive latent traits capturing paternal PND and child development (emotional symptoms, conduct problems, hyperactivity, and peer problems; full details in Supplementary Methods S1). The latent trait approach accounts for measurement error by only modeling shared variance across the items and separating shared from specific variance (57), while also maximizing power by modelling variables as continuous traits (58). The chi-square test of overall fit is prone to model misspecification when the sample size is large (59); thus, we gave preference to relative fit indices, the Root Mean Square Error of Approximation (RMSEA; <0.06), and Comparative Fit Index and Tucker–Lewis Index (CFI/TLI; >0.95), to evaluate the fit of the models (60). The CFA models capturing paternal parenting dimensions, paternal PND, and child development showed an adequate model fit supporting further tests of structural paths (estimates are fully described in Supplementary Methods S1).

### Total, direct, and indirect effects

First, we examined descriptive characteristics of the study sample, including distribution of sociodemographic characteristics by the completeness of data. Second, we examined associations between paternal PND and child emotional symptoms, conduct problems, hyperactivity, and peer problems (total effects). Finally, we examined the extent to which the associations between paternal PND and child emotional and behavioral development are mediated (direct and indirect effects) by three dimensions of paternal involvement. We approached adjustment for confounding variables in steps. First, we estimated the unadjusted models comprising exposure, outcome, and mediators only. Second, we estimated models adjusted for baseline confounders pertaining to socioeconomic, familial, parental characteristics, and child sex (Adjusted^1^). Last, we adjusted analyses for maternal PND, as one of the potentially strongest confounders, to discern its effects on the model estimates (Adjusted^2^). Indirect effects [95% CIs] were calculated using the product-of-coefficients method and bias-corrected (BC) bootstrapping (*n* = 1,000 replications) to account for the non-normal distribution of the outcomes (61). Results from path analyses with continuous scores (latent traits capturing child emotional symptoms, conduct problems, hyperactivity, and peer problems), including indirect effects, are presented as standardized regression coefficients (β). We used MODEL INDIRECT (complete case analyses; *n* = 4,898) and MODEL CONSTRAINT (imputed analyses; *n* = 9,628) commands to estimate total, direct, total, and specific indirect effects with a WLSMV estimator to model continuous (exposure, mediators, and outcomes) and categorical (individual categorical items comprising latent factors) variables. All analyses were conducted using Structural Equation Modelling (SEM) in M*plus* v.8.3 (62).

### Missing data: multiple imputation

Similar to other population-based birth cohorts, ALSPAC is characterized by loss of data due to loss to follow-up. To examine the impact of missing data on our findings, we conducted sensitivity analyses using Multivariate Imputation by Chained Equations (MICE; 63). Imputation methods are fully described in Supplementary Methods S1. Due to relatively high rates of attrition and potential bias associated with including only complete cases in the analysis, as well as loss of precision and power (64), we report models based on the analysis with imputed data, with results of the complete case analysis fully presented in Supplementary Results S1 Tables S3–S6.

## Results

### Study sample characteristics

Never married fathers, those with higher levels of inter-parental conflict and financial difficulties, and those residing in rented accommodation were more likely to experience PND (Table 1). Fathers whose female partners reported higher levels of PND also reported higher PND mean scores. Children of those fathers who reported experiencing higher levels of PND had higher mean emotional symptoms, conduct problems, hyperactivity, and peer problems scores. Distribution of sociodemographic characteristics in the original ALSPAC cohort and the study complete and imputed samples are presented in Table 2. Participants comprising the complete sample were from a higher socioeconomic background, as indexed by a lower proportion of those reporting manual social class and financial difficulties and a higher proportion of those reporting residing in semi-detached/terraced accommodation and married marital status, compared to the original ALSPAC and imputed samples.

**Table 1:** Characteristics of the sample and child emotional and behavioral development (mean scores for emotional symptoms, conduct problems, hyperactivity, and peer problems at 7 years) by the exposure status (paternal PND at 8 months; threshold ≥12).

Exposure status:	Paternal postnatal depression (8 months)	
	No	Yes
	*N* (%)	*N* (%)
	6,713 (95.9)	283 (4.1)
Child and baseline confounders		
Child sex		
Male	3,441 (51.3)	136 (48.1)
Female	3,266 (48.7)	147 (51.9)
Chi^2^, *p*-value		1.15, 0.284
Marital status		
Never married	1,109 (16.8)	74 (27.0)
Married	5,472 (83.2)	200 (73.0)
Chi^2^, *p*-value		19.00, <.001
Paternal educational attainment		
A-Level/Degree	3,310 (54.6)	121 (50.8)
Minimal education/None/O-Level	2,754 (45.4)	117 (49.2)
Chi^2^, *p*-value		1.29, 0.255
Paternal social class		
Non-manual	3,803 (62.1)	138 (56.8)
Manual	2,318 (37.9)	105 (43.2)
Chi^2^, *p*-value		2.83, 0.093
Financial difficulties		
No financial difficulties	4,705 (73.8)	142 (53.38)
Financial difficulties	1,671 (26.2)	124 (46.62)
Chi^2^, *p*-value		53.93, <.001
Type of main dwelling/accommodation		
Detached house	1,038 (15.9)	32 (11.72)
Semi-detached/terraced house	4,597 (70.5)	180 (65.93)
Rented flat/room	890 (13.6)	61 (22.34)
Chi^2^, *p*-value		17.87, <.001
*Paternal age, mean (SD)*	31.0 (5.5)	31.5 (6.5)
ANOVA, *p*-value		−1.28, 0.201
*Parental antenatal conflict, mean (SD)*	9.5 (1.8)	10.3 (1.7)
ANOVA, *p*-value		7.34, <.001
*Maternal PND at 8 weeks*	5.6 (4.5)	8.6 (5.2)
ANOVA, *p*-value		−10.61, <.001
Child emotional and behavioral difficulties at 7 years		
*Child emotional symptoms*	1.48 (1.7)	1.77 (1.81)
ANOVA, *p*-value		−2.47, 0.016
*Child conduct problems*	1.53 (1.4)	2.14 (1.86)
ANOVA, *p*-value		−6.02, <.001
*Child hyperactivity*	3.27 (2.34)	3.72 (2.59)
ANOVA, *p*-value		−2.73, 0.006
*Child peer problems*	1.00 (1.37)	1.51 (1.70)
ANOVA, *p*-value		−5.23, <.001

*p*-values based on Pearson's Chi-square (Chi^2^) test of association between paternal PND and categorical variables, and ANOVA for differences in means for continuous variables; sample sizes vary due to the differences in data availability on socioeconomic, familial, parental and child characteristics. Paternal PND, paternal postnatal depression; Maternal PND, maternal postnatal depression.

**Table 2:** Distribution of sociodemographic characteristics in the original avon longitudinal study of parents and children (ALSPAC) cohort and the study complete and imputed samples.

Sample sociodemographic characteristics assessed during pregnancy^a^	Core ALSPAC sample (*N* = 14,901)	Complete sample (*N* = 4,094)	Imputed sample (*N* = 9,628)
	(%)	(%)	(%)
Paternal educational attainment			
A-Level/Degree	49.2	58.3	50.0
Minimal education/none/O-Level	50.8	41.5	50.0
Paternal social class			
Non-manual	55.9	67.3	57.9
Manual	44.1	32.7	42.1
Presence of financial difficulties			
Financial difficulties	32.1	23.0	29.7
No financial difficulties	67.9	77.0	70.3
Homeownership status			
Detached	14.5	17.4	15.1
Semi-detached/terraced	66.7	72.0	68.3
Flat/room	18.8	10.6	16.6
Marital status			
Married	74.9	86.7	79.9
Never married	25.1	13.3	20.1
	*Mean* (*SD*)	*Mean* (*SD*)	*Mean* (*SD*)
Parental conflict	10.01 (1.77)	10.31 (1.66)	10.11 (1.73)
Paternal age	30.65 (5.69)	31.41 (5.35)	30.66 (5.66)
Maternal PND	6.05 (4.79)	5.62 (4.41)	5.95 (4.68)

^a^ Additional missing data on demographics: paternal educational attainment missing for 3,258/23.6%; paternal social class missing for 3,032/22.0%; financial difficulties missing for 1,909/14%; homeownership status missing for 1,023/7.4%; marital status missing for 913/6.6%; parental conflict missing for 2,038/14.7%; paternal age missing for 5,773/41.7%; maternal PND missing for 2,275/16.4%. Maternal PND: Maternal postnatal depression.

### Paternal involvement factors: paternal parenting confidence, enjoyment and warmth, and conflictual relationship with child

Full details of the factors capturing paternal parenting confidence, enjoyment and warmth, and conflictual relationship with child are provided below. Associations between paternal involvement factors and aspects of child emotional and behavioral development are fully described in Supplementary Results S1 Table S2.

#### Paternal parenting confidence

11 items relating to paternal feelings of confidence in the parenting role and perceptions of the ability to engage effectively in parenting behaviors (e.g., “partner feels confident with child”, “partner unsure if doing the right thing”, “partner happy with the way he brings up child”) were extracted from paternal self-reported questionnaires administered at 8 weeks, 8 months, 1 year 9 months and 2 years 9 months. Higher factor scores represented higher levels of paternal parenting confidence.

#### Paternal enjoyment and warmth

27 items relating to feelings of enjoyment, affection, love and warmth toward the child (e.g., “partner enjoys child”, “partner feels very close to child”, “child gives great joy”) were extracted from paternal self-reported questionnaires administered at 8 weeks, 8 months, 1 year 9 months, 2 years 9 months and 3 years 11 months. Higher factor scores represented more paternal enjoyment, affection and warmth toward the child.

#### Paternal conflictual relationship with child

19 items relating to conflict, harsh disciplining and irritation with the child (e.g., “child gets on partner's nerves”, “partner dislikes mess surrounding child”, “smacking is the best way to discipline child”) were extracted from paternal self-reported questionnaires administered at 8 weeks, 8 months, 1 year 9 months and 2 years 9 months. Higher factor scores signified lower levels of conflictual parent-child relationship, irritation with the child and less harshness in paternal disciplining.

### Total effects: associations between paternal PND and child development

First, we estimated the associations between paternal PND and child emotional symptoms, conduct problems, hyperactivity, and peer problems (total effects) in the unadjusted models, models adjusted for antenatal baseline socioeconomic, familial, paternal characteristics, and child sex (Adjusted^1^), and models further adjusted for maternal PND (Adjusted^2^). Paternal PND was associated with higher levels of child emotional symptoms, conduct problems, hyperactivity, and peer problems in the unadjusted and Adjusted^1^ models (Table 3). These associations were attenuated in Adjusted^2^ models (emotional symptoms: *β* = 0.060, 95% CI: [0.019, 0.101], *p* = 0.004; hyperactivity: *β* = 0.034, 95% CI: [−0.003, 0.071], *p* = 0.083; peer problems: *β* = 0.080, 95% CI: [0.039, 0.121], *p* ≤ 0.001). The attenuation effect of adjustment for maternal PND was particularly evident for child conduct problems [*β* = 0.032, 95% CI: (−0.009, 0.073), *p* = 0.135], with the evidence of total effect nearing zero. Thus, we limited examination of direct and mediated effects in mediation models to child emotional symptoms, hyperactivity, and peer problems as outcomes.

**Table 3:** Total associations between paternal PND and child emotional symptoms, conduct problems, hyperactivity, and peer problems in imputed sample.

Effect Size^a^	Model estimates (*N* = 9,628)					
	Unadjusted model		Adjusted^1^		Adjusted^2^	
	*β* [95% CI]	*P*-value	β [95% CI]	*P*-value	β [95% CI]	*P*-value
Paternal PND (8 months)						
Child emotional symptoms	0.136 [0.095, 0.177]	≤.001	0.101 [0.060, 0.142]	≤.001	0.060 [0.019, 0.101]	.004
Child conduct problems	0.125 [0.086, 0.164]	≤.001	0.061 [0.020, 0.102]	.003	0.032 [−0.009, 0.073]	.135
Child hyperactivity	0.102 [0.065, 0.139]	≤.001	0.064 [0.027, 0.101]	.001	0.034 [−0.003, 0.071]	.083
Child peer problems	0.161 [0.122, 0.200]	≤.001	0.114 [0.075, 0.153]	≤.001	0.080 [0.039, 0.121]	≤.001

^a^ Effect sizes are unadjusted and adjusted regression coefficients (β standardized); Unadjusted models: exposure and outcome only.

Adjusted^1^: Adjusted for antenatal baseline socioeconomic (paternal social class, income and type of accommodation), familial (marital status and parental conflict), paternal (age and education) characteristics and child sex; Adjusted^2^: Further adjusted for maternal PND (8 weeks).

Paternal PND, paternal postnatal depression; Maternal PND, maternal postnatal depression.

### Direct and indirect effects: associations between paternal PND, paternal involvement, and child development

#### Associations between paternal PND (exposure) and paternal parenting confidence, enjoyment and warmth, and conflictual relationship with child (mediators)

Paternal PND was strongly associated with less paternal enjoyment and warmth, more father-child conflict, and less paternal parenting confidence in the unadjusted, Adjusted^1^, and Adjusted^2^ models (paternal enjoyment and warmth: *β* = −0.336, 95% CI: [−0.369, −0.303], *p* ≤ 0.001; father-child conflict: *β* = −0.407, 95% CI: [−0.436, −0.378], *p* ≤ 0.001; paternal parenting confidence: *β* = −0.424, 95% CI: [−0.459, −0.389], *p* ≤ 0.001; see Table 4 for full results).

**Table 4:** Associations between paternal PND and paternal parenting confidence, enjoyment and warmth, and conflictual relationship with child in imputed sample.

Effect Size^a^	Model estimates (*N* = 9,628)					
	Unadjusted model		Adjusted^1^		Adjusted^2^	
	β [95% CI]	*P*-value	β [95% CI]	*P*-value	β [95% CI]	*P*-value
Paternal PND (8 months)						
Paternal enjoyment and warmth	−0.375 [−0.406, −0.344]	≤.001	−0.359 [−0.390, −0.328]	≤.001	−0.336 [−0.369, −0.303]	≤.001
Paternal conflictual relationship with child	−0.464 [−0.493, −0.435]	≤.001	−0.438 [−0.465, −0.410]	≤.001	−0.407 [−0.436, −0.378]	≤.001
Paternal parenting confidence	−0.467 [−0.502, −0.432]	≤.001	−0.453 [−0.488, −0.418]	≤.001	−0.424 [−0.459, −0.389]	≤.001

^a^ Effect size are unadjusted and adjusted regression coefficients (β standardized); Unadjusted models: exposure and outcome only.

Adjusted^1^: Adjusted for antenatal baseline socioeconomic (paternal social class, income and type of accommodation), familial (marital status and parental conflict), paternal (age and education) characteristics and child sex; Adjusted^2^: Further adjusted for maternal PND (8 weeks).

Paternal PND, paternal postnatal depression; Maternal PND, maternal postnatal depression.

#### Associations between paternal parenting confidence, enjoyment and warmth, conflictual relationship with child (mediators) and child emotional symptoms, hyperactivity, and peer problems (outcomes)

Higher levels of paternal parenting confidence and lower levels of father-child conflict were associated with lower levels of child emotional symptoms and hyperactivity in the unadjusted, Adjusted^1^, and Adjusted^2^ models (paternal parenting confidence to child emotional symptoms: *β* = −0.154, 95% CI: [−0.240, −0.068], *p* ≤ 0.001; paternal parenting confidence to child hyperactivity: *β* = −0.085, 95% CI: [−0.151, −0.018], *p* = 0.013; father-child conflict to child emotional symptoms: *β* = −0.103, 95% CI: [−0.170, −0.036], *p* = 0.002; father-child conflict to child hyperactivity: *β* = −0.125, 95% CI: [−0.183, −0.066], *p* ≤ 0.001; see Table 5 for full results). Higher levels of paternal parenting confidence were associated with lower risk of child peer problems in the unadjusted, Adjusted^1^, and Adjusted^2^ models, albeit with wide 95% CIs [*β* = −0.099, 95% CI: (−0.195, −0.003), *p* = 0.043]. There was no evidence for the association between father-child conflict and child peer problems in the unadjusted, Adjusted^1^, and Adjusted^2^ models.

**Table 5:** Associations between paternal parenting confidence, enjoyment and warmth, and conflictual relationship with child and child emotional symptoms, hyperactivity, and peer problems in imputed sample.

Effect Size^a^	Model estimates (*N* = 9,628)					
	Unadjusted model		Adjusted^1^		Adjusted^2^	
	β [95% CI]	*P*-value	β [95% CI]	*P*-value	β [95% CI]	*P*-value
Child emotional symptoms						
Paternal enjoyment and warmth	0.091 [0.024, 0.158]	.007	0.077 [0.010, 0.144]	.022	0.074 [0.009, 0.139]	.025
Paternal conflictual relationship with child	−0.127 [−0.194, −0.060]	≤.001	−0.122 [−0.191, −0.053]	≤.001	−0.103 [−0.170, −0.036]	.002
Paternal parenting confidence	−0.168 [−0.254, −0.082]	≤.001	−0.168 [−0.256, −0.080]	≤.001	−0.154 [−0.240, −0.068]	≤.001
Child hyperactivity						
Paternal enjoyment and warmth	0.045 [−0.018, 0.108]	.155	0.043 [−0.018, 0.104]	.160	0.041 [−0.018, 0.100]	.181
Paternal conflictual relationship with child	−0.138 [−0.197, −0.079]	≤.001	−0.138 [−0.200, −0.080]	≤.001	−0.125 [−0.183, −0.066]	≤.001
Paternal parenting confidence	−0.087 [−0.156, −0.018]	.014	−0.095 [−0.164, −0.026]	.006	−0.085 [−0.151, −0.018]	.013
Child peer problems						
Paternal enjoyment and warmth	0.037 [−0.041, 0.115]	.348	0.025 [−0.051, 0.101]	.519	0.022 [−0.054, 0.010]	.580
Paternal conflictual relationship with child	−0.053 [−0.125, 0.019]	.148	−0.054 [−0.126, 0.018]	.148	−0.038 [−0.108, 0.032]	.304
Paternal parenting confidence	−0.106 [−0.202, −0.010]	.032	−0.110 [−0.206, −0.014]	.025	−0.099 [−0.195, −0.003]	.043

^a^ Effect size are unadjusted and adjusted regression coefficients (β standardized); Unadjusted models: exposure and outcome only.

Adjusted^1^: Adjusted for antenatal baseline socioeconomic (paternal social class, income and type of accommodation), familial (marital status and parental conflict), paternal (age and education) characteristics and child sex; Adjusted^2^: Further adjusted for maternal PND (8 weeks).

Paternal PND, paternal postnatal depression; Maternal PND, maternal postnatal depression.

No evidence for associations between paternal enjoyment and warmth and child hyperactivity and peer problems emerged in the unadjusted, Adjusted^1^, and Adjusted^2^ models. There was evidence of positive association between paternal enjoyment and warmth and child emotional symptoms [Adjusted^2^: *β* = 0.074, 95% CI: (0.009, 0.139), *p* = 0.025; Table 5], suggesting that higher levels of paternal enjoyment and warmth are associated with higher levels of adverse child emotional development. We conducted additional sensitivity analyses to examine the nature of the associations between dimensions of paternal involvement and child emotional symptoms, hyperactivity, and peer problems independent of adjustment for other paternal involvement factors in the unadjusted, Adjusted^1^, and Adjusted^2^ models (imputed analyses). The sensitivity analyses revealed that, once mutual adjustment for each of the parenting confounders was accounted for, the strength of the association between paternal enjoyment and warmth and child emotional symptoms was considerably weakened [Adjusted^2^: *β* = −0.046, 95% CI: (−0.010, 0.005), *p* = 0.076; Supplementary Table S7] and reversed (i.e., higher levels of paternal enjoyment and warmth associated with lower levels of adverse offspring emotional and behavioral development).

#### Direct and indirect effects

Strong evidence emerged for total indirect effects from paternal PND at 8 months to child emotional symptoms, hyperactivity, and peer problems at age 7 years through the combination of all dimensions of paternal involvement in the unadjusted, Adjusted^1^, and Adjusted^2^ models (emotional symptoms: *β* = 0.083, 95% CI:[ 0.059, 0.106], *p* ≤ 0.001; hyperactivity: *β* = 0.073, 95% CI: [0.051, 0.095], *p* ≤ 0.001; peer problems: *β* = 0.050, 95% CI: [0.023, 0.077], *p* ≤ 0.001; see Table 6 for full results). There was no evidence of direct effects from paternal PND to child emotional symptoms, hyperactivity, and peer problems once the indirect effects via all dimensions of paternal involvement were accounted for.

**Table 6:** Estimates of direct and indirect effects in imputed sample.

Effect Size^a^	Model estimates (*N* = 9,628)					
	Unadjusted model		Adjusted^1^		Adjusted^2^	
	β [95% CI]	*P*-value	β [95% CI]	*P*-value	β [95% CI]	*P*-value
Child emotional symptoms						
1. Total indirect effect	0.104 [0.078, 0.129]	≤.001	0.102 [0.076, 0.127]	≤.001	0.083 [0.059, 0.106]	≤.001
2. Direct effect	0.031 [−0.016, 0.078]	.303	−0.001 [−0.048, 0.046]	.962	−0.023 [−0.068, 0.022]	.320
3. Total effect	0.135 [0.096, 0.174]	≤.001	0.101 [0.060, 0.142]	≤.001	0.060 [0.019, 0.101]	.004
4. Specific indirect effects						
Paternal enjoyment and warmth	−0.034 [−0.059, −0.008]	.008	−0.028 [−0.051, −0.004]	.023	−0.025 [−0.046, −0.003]	.026
Paternal conflictual relationship with child	0.059 [0.028, 0.090]	≤.001	0.054 [0.025, 0.083]	.001	0.042 [0.015, 0.069]	.002
Paternal parenting confidence	0.079 [0.038, 0.120]	≤.001	0.076 [0.035, 0.117]	≤.001	0.065 [0.028, 0.102]	.001
Child hyperactivity						
1. Total indirect effect	0.088 [0.064, 0.111]	≤.001	0.088 [0.064, 0.111]	≤.001	0.073 [0.051, 0.095]	≤.001
2. Direct effect	0.014 [−0.033, 0.061]	.575	−0.024 [−0.071, 0.023]	.316	−0.039 [−0.086, 0.008]	.095
3. Total effect	0.101 [0.064, 0.138]	≤.001	0.064 [0.027, 0.101]	.001	0.034 [−0.003, 0.071]	.081
4. Specific indirect effects						
Paternal enjoyment and warmth	−0.017 [−0.041, 0.006]	.156	−0.015 [−0.037, 0.007]	.161	−0.014 [−0.034, 0.006]	.181
Paternal conflictual relationship with child	0.064 [0.036, 0.091]	≤.001	0.060 [0.035, 0.085]	≤.001	0.051 [0.027, 0.075]	≤.001
Paternal parenting confidence	0.040 [0.007, 0.073]	.015	0.043 [0.012, 0.074]	.007	0.036 [0.007, 0.065]	.014
Child peer problems						
1. Total indirect effect	0.060 [0.031, 0.089]	≤.001	0.064 [0.035, 0.093]	≤.001	0.050 [0.023, 0.077]	≤.001
2. Direct effect	0.099 [0.046, 0.152]	≤.001	0.049 [−0.002, 0.100]	.060	0.029 [−0.022, 0.080]	.250
3. Total effect	0.159 [0.120, 0.198]	≤.001	0.113 [0.073, 0.152]	≤.001	0.079 [0.038, 0.120]	≤.001
4. Specific indirect effects						
Paternal enjoyment and warmth	−0.014 [−0.043, 0.015]	.348	−0.009 [−0.036, 0.018]	.517	−0.007 [−0.032, 0.018]	.578
Paternal conflictual relationship with child	0.025 [−0.008, 0.058]	.151	0.024 [−0.007, 0.055]	.152	0.015 [−0.014, 0.044]	.307
Paternal parenting confidence	0.050 [0.005, 0.095]	.034	0.050 [0.005, 0.095]	.027	0.042 [0.001, 0.083]	.047

^a^ Effect size are unadjusted and adjusted regression coefficients (β standardized); Unadjusted models: exposure and outcome only.

Adjusted^1^: Adjusted for antenatal baseline socioeconomic (paternal social class, income and type of accommodation), familial (marital status and parental conflict), paternal (age and education) characteristics and child sex; Adjusted^2^: Further adjusted for maternal PND (8 weeks).

Paternal PND, paternal postnatal depression; Maternal PND, maternal postnatal depression.

Evidence for specificity of indirect effects emerged depending on the child outcome. Specifically, paternal enjoyment and warmth, father-child conflict, and paternal parenting confidence mediated the association between paternal PND and child emotional symptoms in the unadjusted, Adjusted^1^, and Adjusted^2^ models (paternal enjoyment and warmth: *β* = −0.025, 95% CI: [−0.046, −0.003], *p* = 0.026; father-child conflict: *β* = 0.042, 95% CI: [0.015, 0.069], *p* = 0.002; paternal parenting confidence: *β* = 0.065, 95% CI: [0.028, 0.102], *p* = 0.001). There was evidence that father-child conflict and paternal parenting confidence mediated the association between paternal PND and child hyperactivity in the unadjusted, Adjusted^1^, and Adjusted^2^ models (father-child conflict: *β* = 0.051, 95% CI: [0.027, 0.075], *p* ≤ 0.001; paternal parenting confidence: *β* = 0.036, 95% CI: [0.007, 0.065], *p* = 0.014). There was some evidence that paternal parenting confidence mediated the association between paternal PND and child peer problems in the unadjusted, Adjusted^1^, and Adjusted^2^ models; however, the 95% CIs were wide [*β* = 0.042, 95% CI: (0.001, 0.083), *p* = 0.047]. There was no evidence for specific indirect effects through paternal enjoyment and warmth in the association between paternal PND and child hyperactivity and peer problems in unadjusted, Adjusted^1^, and Adjusted^2^ models. When the analyses were repeated using the sample with complete data, estimates of the total, direct, and indirect effects were in the same direction as they had been in the imputed data analyses, leading to the same overarching conclusions. The substantially reduced sample size, however, led to insufficient statistical power to detect some of the total and specific indirect effects in complete case analyses (estimates are fully described in Supplementary Results S1 and Tables S3–S6).

## Discussion

### Main findings

In this large population-based birth cohort, we used longitudinal data to estimate the extents to which associations between paternal PND at 8 months and child emotional symptoms, conduct problems, hyperactivity, and peer problems at 7 years are mediated by paternal parenting confidence, enjoyment and warmth, and conflictual father-child relationship in early childhood.

There was evidence of total associations between paternal PND and higher levels of child emotional symptoms, conduct problems, hyperactivity and peer problems, which were substantially attenuated once all child, family, socioeconomic and parental characteristics were accounted for. Attenuation was particularly evident following adjustment for maternal PND, and for child conduct problems, with the total effect nearing zero. These findings are consistent with a meta-analysis reporting weak to moderate longitudinal associations between paternal PND and child emotional and behavioral outcomes (7), including increased levels of child peer problems (65).

Evidence regarding associations between paternal PND and child externalizing behaviors, including conduct problems, is mixed. Two recent meta-analyses found small but consistent associations between paternal PND and child externalizing behaviors (8, 66), while other studies find no such effects or show weak negative associations (67, 68). Maternal PND and parental conflict may be stronger predictors of child externalizing difficulties than paternal PND (67, 69), which is reflected in our findings indicating substantial attenuation between paternal PND and child behavioral problems and hyperactivity after accounting for these factors.

One of the strengths of our study is that we examine specificity, both in risks and offspring outcomes, when considering paternal involvement in the context of paternal PND (34). Consistent with existing research, paternal PND was strongly associated with less paternal parenting confidence, less paternal enjoyment and warmth, and more father-child conflict (16). We also observed differential patterns of associations between dimensions of paternal involvement and child emotional and behavioral development, with higher levels of paternal parenting confidence being associated with lower levels of child emotional symptoms, hyperactivity, and peer problems. These findings align with the meta-analysis of the Positive Parenting Programme (Triple P), suggesting that improving parenting efficacy and confidence is the key mechanism for preventing child emotional and behavioral problems (70). Family systems interventions also emphasize the importance of providing support at the family-level to enhance parental parenting competencies, which enable parent-child interactions conducive of more optimal child development (71). Family systems framework may be particularly important in the context of parental PND, given strong associations between maternal and paternal PND (53, 54) and the adverse impacts that depression exerts on both parents' parenting (14, 16).

Positive and less conflictual father-child relationships are protective factors for less adverse child development (72). By contrast, conflictual father-child relationships are a common vulnerability that increases the risk for multiple internalizing and externalizing child disorders (73). Consistently, our findings support lower levels of child emotional symptoms in the context of less conflictual father-child relationships, which were also independently associated with lower levels of child hyperactivity. We found no evidence for the associations between paternal enjoyment and warmth and child hyperactivity and peer problems. It may be that our self-report measure did not capture key aspects of paternal enjoyment, warmth, and sensitivity that are important for child emotional and behavioral development (74). Arguably, parental self-reports of warmth and sensitivity toward the child may be biased, particularly in the context of parental depression or misinterpretation of child behavior (75). Future research may benefit from examining nuanced variations in these complex parental behaviors and their impact on child development using direct observations of father-child interactions. Conceptually, the evidence regarding associations between parental sensitivity and child behavioral development remains inconsistent, with studies reporting associations of varying magnitude (76). Further research is also needed to determine how variations among children and their circumstances influence the strength of these associations, with important research and practice implications. Theoretically and empirically, there may also be distinct causal influences of paternal warmth and enjoyment, as a proxy measure of paternal sensitivity, on emotional and behavioral dimensions of child development (77, 78), warranting investigation beyond the scope of this study.

### Direct and indirect effects

Associations between paternal PND and child emotional symptoms, hyperactivity, and peer problems at age 7 years were explained by the combination of all dimensions of paternal involvement, including enjoyment and warmth, parenting confidence, and conflictual relationship with child. A previous ALSPAC-based study found that a relatively small proportion of the association between paternal PND and child emotional and behavioral development at ages 4 and 7 years was mediated by paternal non-involvement (analyzed as a sum-score), with maternal depression and parental conflict contributing to the majority of the mediation effect (19). However, measures of paternal non-involvement and child outcomes were based on maternal reports potentially underestimating the strength of the indirect effect through paternal parenting.

We found evidence for specificity of indirect effects depending on child outcome, with less paternal parenting confidence and more conflictual father-child relationship mediating the association between paternal PND, child emotional symptoms, and hyperactivity. Lower levels of paternal parenting confidence also mediated the association between paternal PND and child peer problems, although 95% CIs for the effect were wide. The likely negative impact of paternal PND on fathers' perceptions of their own competence in the parenting role may undermine their ability to be responsive to their children (79), display affectionate and supportive behavior (80), engage in high-quality interactions (81), and effective emotion regulation (82), resulting in increased levels of child emotional symptoms, hyperactivity, and peer problems (30). Father-child conflict also mediates the association between paternal PND and child emotional and behavioral development, including emotion regulation problems (20, 83). These findings suggest that paternal parenting confidence and father-child conflict may be important prevention and intervention targets to reduce adverse emotional and behavioral outcomes in children of fathers experiencing PND (30, 84).

Our findings indicated that higher levels of paternal enjoyment and warmth were associated with higher levels of adverse child emotional development. In order to substantiate these findings, we conducted further sensitivity analyses to examine associations between dimensions of paternal involvement and child emotional symptoms, hyperactivity and peer problems independent of adjustment for other paternal involvement factors. In these analyses, there was some evidence that higher levels of paternal enjoyment and warmth were associated with lower levels of adverse child development, albeit with wide 95% CIs in the fully adjusted models, suggesting that mutual adjustment for each of the parenting factors in the main mediation model may have weakened, and potentially reversed, these associations further. Therefore, the findings regarding inconsistent direction of associations between paternal enjoyment and warmth and child emotional development warrant a cautious interpretation as they may represent a potential statistical artifact resulting from the complexity of our multiple-mediator model.

### Strengths and limitations

The current study has several strengths. First, ALSPAC is a unique intergenerational birth-cohort study with a large and overall representative sample of UK fathers (37). The availability of rich repeated self-reported measures of paternal parenting enabled us to model several cognitive and affective/behavioral dimensions of paternal involvement using the latent factor models across multiple time points in early childhood, capturing the longitudinal nature of paternal parenting. Nevertheless, cautious generalization of our findings to contemporary families is needed. The ALSPAC cohort, born between 1990 and 1991, reflects limited racial diversity and was largely composed of nuclear families. Parenting behaviors are known to vary across cultural contexts and may be perceived and enacted differently depending on cultural norms (85). Furthermore, family structures and gender dynamics in parenting have continued to evolve, suggesting that our findings may not fully capture the complexity of contemporary family life. Applying the latent factor approach also enabled us to model child development as four different dimensions (i.e., emotional symptoms, conduct problems, hyperactivity, and peer problems), rather than a sum score, to examine potential differential associations between paternal PND, paternal involvement, and child development in line with the specificity principle in developmental research (34).

Second, we examined possible roles of these dimensions in transmitting the risk of paternal PND on child emotional and behavioral development in mediation models adjusted for a range of socioeconomic, family, parental, and child characteristics, including parental conflict and maternal PND. There continues to be lack of studies that examine potential mediating roles of multiple dimensions of paternal involvement in the association between paternal PND and different aspects of child emotional and behavioral development (7, 19), with our study representing a potentially important corrective.

Third, although the exposure (paternal PND) and mediators (paternal involvement) were measured using paternal self-reports, the outcomes (child emotional and behavioral development) were reported by the mothers, strengthening study findings by reducing the risk of shared variance bias and inflated estimates of total associations.

Still, there are limitations to this study. We did not include additional assessments of maternal and paternal PND later in development due to moderate-to-high correlations between time-points (42) and increased risk of overadjustment bias (86). Previous ALSPAC studies (42) reported evidence of stability and predictive validity of paternal PND across assessments, indicating that fathers who experienced depression during pregnancy and the early postnatal period were also more likely to report depression at later assessments. Paternal depression at later time points is also more likely to be on a causal pathway, explaining rather than confounding, the association between paternal PND and child development (later paternal depression may be a consequence of lack of paternal involvement). However, epidemiological evidence indicates that paternal depression likely recurs across development (3, 6), thus, estimating associations between paternal depression at one time-point (8 months postnatally) and child emotional and behavioral development may represent a limitation of our conceptual approach.

Some paternal involvement items were measured concurrently with the assessment of paternal PND (8 months), providing an alternative explanation whereby lower paternal involvement is associated with higher risk of paternal PND. However, existing evidence strongly supports the adverse effect of paternal PND on paternal parenting and involvement (16), suggesting that these associations are unlikely to be in the opposite direction. Nevertheless, this possibility cannot be fully discarded and needs to be taken into account when interpreting our findings.

Even though our sample attrition is similar to that observed in other population-based studies (38, 39), it is a limitation with potential implications for internal validity. We addressed bias due to selective attrition (fathers from lower socioeconomic background and those experiencing depression were under-represented in complete case sample) by controlling for factors known to predict missingness and by imputing missing data in the exposure, outcomes and confounders. The conclusions from the imputed and complete data analyses were the same, with some evidence of stronger total and specific indirect effects in imputed analyses, suggesting that attrition may have contributed to the under- not over-estimation of effects.

Another limitation is lack of independently assessed measures of paternal involvement, which may potentially lead to overestimation of the indirect effects due to shared-method bias (e.g., fathers' depressive symptoms may negatively bias self-reports of parenting behaviors; 16). Although social desirability bias (87) may affect both self-reported and independently observed measures of parenting (88), it may be more pronounced in observational methods (16). We were unable to substantiate self-reported measures of paternal involvement with comparable domains of independently observed data as they were not collected in the ALSPAC cohort. Examination of genetic confounding that may explain associations between paternal PND and child emotional and behavioral development warrants further attention (89) but was beyond the scope of this study, while residual confounding also remains a possibility (90). Replication with a more ethnically and culturally diverse sample of fathers reflecting contemporary paternal parenting practices, as well as examination of child and parent sex effects on parental PND and parenting is also warranted.

### Implications for practice

Our findings contribute to a growing body of epidemiological evidence indicating that paternal PND is a risk factor for adverse child emotional and behavioral development (7), with lower levels of paternal parenting confidence and higher levels of father-child conflict playing important roles in explaining these negative effects. Our study highlights the importance of previously articulated implications, including the needs to (1) recognize (extending screening for depression to both mothers and fathers) and treat depression in both parents when one parent experiences depression (91); (2) design intervention and prevention programs that improve both parents' relationships with the child to facilitate healthy child development (14); and (3) incorporate assessment of the family environment and functioning (e.g., parental conflict) into prevention and intervention programs to strengthen family relationships within the family unit in contradiction to the current focus on one parent (92). However, in practice, a qualitative meta-synthesis of the experiences of fathers suggests that they continue to be marginalized and neglected in the context of perinatal services delivery (93), while existing parenting programs fail to successfully engage fathers (94) even though there is evidence that paternal parenting may be positively influenced by interventions (95), including enhancing paternal sensitive parenting through prenatal video-feedback (96). Our findings may contribute to an increasing shift in thinking about addressing intergenerational transmission of emotional and behavioral risks in families through focusing on both parents' mental health, parenting, and contributions to the family environment.

## Data Availability

ALSPAC data are available through a system of managed open access. The study website contains details of all the data that is available through a fully searchable data dictionary and variable search tool data dictionary. The application steps for ALSPAC data access are highlighted below. 1. Please read the ALSPAC access policy, which describes the process of accessing the data in detail, and outlines the costs associated with doing so. 2. You may also find it useful to browse the fully searchable research proposals database, which lists all research projects that have been approved since April 2011. 3. Please submit your research proposal for consideration by the ALSPAC Executive Committee. You will receive a response within 10 working days to advise you whether your proposal has been approved. If you have any questions about accessing data, please email alspac-data@bristol.ac.uk. Requests to access the datasets should be directed to alspac-data@bristol.ac.uk.
