# Supplementary material for: Paternal postnatal depression and child development at age 7 years in a UK-birth cohort: the mediating roles of paternal parenting confidence, warmth, and conflict
Source: Front Child Adolesc Psychiatry. 2025 Sep 12;4:1650799. doi: 10.3389/frcha.2025.1650799 (PMC12463979; doi:10.3389/frcha.2025.1650799)
Supplement: Supplementary file 1 [file Supplementaryfile1.docx]

**Paternal Postnatal Depression and Child Development at Age 7 Years in a UK-Birth Cohort: The Mediating Roles of Paternal Parenting Confidence, Warmth, and Conflict**

**Supplementary Material**

**Methods**

**Statistical Analyses**

**Latent Factor Models**

In order to identify the CFA models capturing paternal parenting dimensions, paternal PND, and child development, the mean of the latent factors was fixed to ‘0’ and variances to ‘1’. The CFA models capturing paternal PND (RMSEA: 0.043, 95%CI: [0.040 to 0.047]; CFI/TLI: 0.977/0.969), parenting confidence, enjoyment, and warmth, conflictual father-child relationship (RMSEA: 0.018; 95%CI: [0.018 to 0.019]; CFI/TLI: 0.954/0.948), and emotional symptoms, conduct problems, hyperactivity and peer problems in childhood (RMSEA: 0.018; 95%CI: [0.015 to 0.021]; CFI/TLI: 0.990/0.984) showed an adequate model fit supporting further tests of structural paths (i.e., total, direct, and indirect effects).

**Missing Data: Multiple Imputation**

We used Multivariate Imputation by Chained Equations (MICE; Royston & White, 2011) to impute missing data in exposure (paternal PND), outcome (child motional and behavioural development) and confounders (socioeconomic, family, parental, and child characteristics) using the *ice* command in Stata v.15.1/MP (Stata.Corp., Texas, USA). ALSPAC provides a wealth of rich, prospectively collected data on a range of sociodemographic, parental and child mental health, as well as developmental variables, which enabled us to account for missing data in exposure, outcome, confounders, and factors that explain missingness to validate the Missing-At-Random assumption (MAR; White et al., 2011). We took a decision not to impute mediators (affective and cognitive dimensions of paternal involvement) as there are no sufficient auxiliary data on fathers’ parenting in the ALSPAC cohort to justify the plausibility of MAR assumption. The imputation model was fully compatible with the complete case main analyses. Using binary, ordinal logistic, and linear regression models as appropriate, 50 imputed datasets by 10 cycles of regression switching were generated. Monte-Carlo errors were less than 10% of the standard error and FMI values were no larger than 0.5, suggesting that 50 imputed datasets were sufficient (White et al., 2011). Each imputation model contained all variables in the substantive analyses along with over 50 auxiliary variables pertaining to maternal and paternal characteristics (e.g., smoking and drinking) and psychopathology (e.g., repeated EPDS scores assessed at different time points), child development and mental health, as well maternal and paternal indices of socioeconomic adversity (e.g., income). MICE imputations were carried out in Stata v.15.1/MP (Stata.Corp., Texas, USA). The imputed datasets (n=9,628) were exported into M*plus* v.8.3 (Muthén & Muthén, 2015) to estimate direct and indirect (mediated) pathways.

**Results**

**Associations Between Paternal Involvement Factors and Aspects of Child Emotional and Behavioral Development**

Higher levels of paternal parenting confidence were strongly associated with lower levels of conflictual father-child relationship and higher levels of paternal enjoyment and warmth toward the child, while lower levels of conflictual relationship with child were associated with higher levels of paternal enjoyment and warmth (Table S2).

Lower levels of child emotional symptoms were associated with lower levels of child conduct problems, hyperactivity, and peer problems (Table S2). In addition, lower levels of child conduct problems were associated with lower levels of child hyperactivity and peer problems, while lower levels of child hyperactivity were associated with lower levels of peer problems.

**Complete Case Analyses**

When the analyses were repeated using the sample with complete data, estimates of the total, direct and indirect effects were in the same direction as they had been in the imputed data analyses and led to the same overarching conclusions. The substantially reduced sample size, however, led to insufficient statistical power to detect some of the total and specific indirect effects in complete case analyses. Specifically, the association between paternal PND and higher levels of child emotional symptoms was substantially attenuated in the Adjusted^2^ model (β=0.032, 95% CI: [-0.017, 0.081], *p*=0.189; Table S3), with weaker evidence for the association between paternal PND and higher levels of child peer problems (Adjusted^2^: β=0.057, 95% CI: [0.004, 0.110], *p*=0.034). In comparison to imputed data analyses, there was stronger evidence for the association between paternal PND and higher levels of child hyperactivity in the Adjusted^2^ model (β=0.048, 95% CI: [0.003, 0.093], *p*=0.035). Similar to the imputed data analyses, the association between paternal PND and higher levels of child conduct problems was substantially attenuated in the Adjusted^2^ model (β=0.012, 95% CI: [-0.039, 0.063], *p*=0.654).

The associations between paternal PND and paternal parenting confidence, enjoyment and warmth, and conflictual relationship with child were comparable, if somewhat stronger, with imputed data analyses (see Table S4 for full results). The pattern of associations between dimensions of paternal involvement and child emotional and behavioral development in imputed and complete case analyses was broadly similar, with few notable distinctions. In particular, the association between lower levels of father-child conflict and lower risk of child emotional symptoms was substantially attenuated in complete case analyses (Adjusted^2^: β=-0.062, 95% CI: [-0.156, 0.032], *p*=0.195; see Table S5 for full results). There was weaker evidence for the association between paternal enjoyment and warmth and child emotional symptoms in the Adjusted^2^ model (β=0.072, 95% CI: [-0.014, 0.158], *p*=0.102) as indicated by wider 95% CIs and higher p-value. In addition, the association between higher levels of paternal parenting confidence and lower risk of child peer problems was substantially attenuated in the Adjusted^2^ model (β=-0.063, 95% CI: [-0.174, 0.049], *p*=0.269).

Complete case analyses also indicated weaker evidence for specificity through certain indirect effects. In particular, there was weaker evidence that father-child conflict mediated the association between paternal PND and child emotional symptoms in the Adjusted^2^ models (β=0.027, 95% CI: [-0.014, 0.068], *p*=0.197; Table S6). Although the magnitude of specific indirect effect through paternal enjoyment and warmth in the Adjusted^2^ model (β=-0.025, 95% CI: [-0.046, -0.003], *p*=0.026) was similar in complete and imputed data analyses, the 95%CIs were wider in the latter. In contrast to imputed data analyses, there was no evidence that paternal parenting confidence mediated the association between paternal PND and child problems in complete case analyses (Adjusted^2^: β=0.028, 95% CI: [-0.023, 0.079], *p*=0.268).

**References**

Muthén, L. K., & Muthén, B. O. (2015). *Mplus User’s Guide*, 7^th^ ed. Muthén & Muthén: Los Angeles, CA.

Royston, P., & White, I. R. (2011). Multiple imputation by chained equations (MICE): implementation in Stata. *Journal of Statistical Software*, *45*(4), 1-20.

Schisterman, E. F., Cole, S. R., & Platt, R. W. (2009). Overadjustment bias and unnecessary adjustment in epidemiologic studies. *Epidemiology*, *20*(4), 488-495. doi: [10.1097/EDE.0b013e3181a819a1](https://doi.org/10.1097%2FEDE.0b013e3181a819a1)

White, I. R., Royston, P., & Wood, A. M. (2011). Multiple imputation using chained equations: issues and guidance for practice. *Statistics in Medicine, 30*(4), 377-99.

<https://doi.org/10.1002/sim.4067>

**Table S1.** Derived factors, individual parenting items, age at assessment and standardised factor loadings.

| Item | Age at assessment/Factor | Item description | Factor loading | *SE* |
| --- | --- | --- | --- | --- |
|  | Paternal parenting confidence | | | |
| 1. | 8 weeks | Partner feels confident with child | 0.436 | 0.027 |
| 2. | 8 weeks | Partner happy with the way he brings up child | 0.568 | 0.027 |
| 3. | 8 weeks | Partner regrets lack of experience with child | 0.319 | 0.035 |
| 4. | 8 weeks | Partner feels well-prepared for birth and childcare | 0.367 | 0.024 |
| 5. | 8 weeks | Having a baby is as expected | 0.328 | 0.024 |
| 6. | 8 months | Partner feels confident with child | 0.645 | 0.026 |
| 7. | 8 months | Partner unsure whether doing the right thing | 0.498 | 0.027 |
| 8. | 1 year 9 months | Partner afraid to be alone with toddler | 0.454 | 0.081 |
| 9. | 1 year 9 months | Partner sure doing the right thing for child | 0.529 | 0.027 |
| 10. | 2 years 9 months | Partner feels confident with child | 0.750 | 0.029 |
| 11. | 2 years 9 months | Partner feels constantly unsure if doing right thing for child | 0.508 | 0.028 |
|  | Paternal enjoyment and warmth | | | |
| 1. | 8 weeks | Partner is making a strong bond with child | 0.515 | 0.013 |
| 2. | 8 weeks | Partner feels guilty at not enjoying child | 0.630 | 0.022 |
| 3. | 8 weeks | Child made partner more fulfilled | 0.442 | 0.020 |
| 4. | 8 months | Partner enjoys child | 0.692 | 0.020 |
| 5. | 8 months | Partner preferred not to have had child | 0.568 | 0.026 |
| 6. | 8 months | Pleasure watching child develop | 0.725 | 0.022 |
| 7. | 8 months | Partner feels should enjoy child, but they are not | 0.702 | 0.022 |
| 8. | 8 months | Child made partner more fulfilled | 0.547 | 0.019 |
| 9. | 8 months | Partner feels children are fun | 0.545 | 0.02 |
| 10. | 8 months | Partner enjoys seeing child after work | 0.664 | 0.022 |
| 11. | 8 months | Partners feels having child made more fulfilled | 0.515 | 0.025 |
| 12. | 8 months | Talking to child is important | 0.384 | 0.047 |
| 13. | 8 months | Cuddling child is very important | 0.310 | 0.058 |
| 14. | 1 year 9 months | Children are fun | 0.490 | 0.024 |
| 15. | 1 year 9 months | Partner really loves child | 0.629 | 0.031 |
| 16. | 1 year 9 months | Partner glad they had child | 0.596 | 0.027 |
| 17. | 1 year 9 months | Partner feels great pleasure watching child grow | 0.662 | 0.026 |
| 18. | 1 year 9 months | Child gives great joy | 0.665 | 0.025 |
| 19. | 2 years 9 months | Partner really enjoys child | 0.660 | 0.022 |
| 20. | 2 years 9 months | Partner feels great pleasure watching child develop | 0.701 | 0.026 |
| 21. | 2 years 9 months | Partner feels should enjoy child, but they are not | 0.628 | 0.025 |
| 22. | 2 years 9 months | Partners feels having child made more fulfilled | 0.431 | 0.026 |
| 23. | 2 years 9 months | Partner enjoys seeing child after work | 0.623 | 0.024 |
| 24. | 3 years 11 months | Child makes partner feel very happy | 0.342 | 0.079 |
| 25. | 3 years 11 months | Partner feels very close to child | 0.422 | 0.045 |
| 26. | 3 years 11 months | Child is very affectionate to partner | 0.428 | 0.055 |
| 27. | 3 years 11 months | Cuddling is best way to calm child | 0.643 | 0.015 |
|  | Paternal conflictual relationship with child | | | |
| 1. | 8 weeks | Partner so stressed at home it is a bad influence on child | 0.546 | 0.026 |
| 2. | 8 weeks | Partner regrets having child | 0.606 | 0.034 |
| 3. | 8 months | Partner dislikes mess surrounding child | 0.574 | 0.022 |
| 4. | 8 months | Partner finds child crying unbearable | 0.661 | 0.021 |
| 5. | 8 months | Partner feels they have no time to themselves | 0.651 | 0.023 |
| 6. | 1 year 9 months | Child whining can make parent want to hit | 0.489 | 0.025 |
| 7. | 1 year 9 months | Having young child is absolutely exhausting | 0.392 | 0.026 |
| 8. | 1 year 9 months | Smacking is the best way to discipline child | 0.185 | 0.029 |
| 9. | 1 year 9 months | Parent can feel exasperated calming child | 0.327 | 0.023 |
| 10. | 1 year 9 months | Child never gets on partner’s nerves | 0.274 | 0.022 |
| 11. | 1 year 9 months | Partner cannot bear it when child cries | 0.353 | 0.025 |
| 12. | 1 year 9 months | Partner feels desperate when child complains | 0.576 | 0.024 |
| 13. | 1 year 9 months | Child demands bring intense anger | 0.558 | 0.026 |
| 14. | 2 years 9 months | Partner dislikes mess surrounding child | 0.546 | 0.024 |
| 15. | 2 years 9 months | Partner cannot bear it when child cries | 0.518 | 0.025 |
| 16. | 2 years 9 months | Partner feels they have no time to themselves | 0.539 | 0.027 |
| 17. | 2 years 9 months | Partner often gets very irritated with child | 0.544 | 0.027 |
| 18. | 2 years 9 months | Partner has frequent battles of will with child | 0.391 | 0.028 |
| 19. | 2 years 9 months | Child gets on partner’s nerves | 0.420 | 0.035 |

**Table S2.** Associations between paternal involvement factors and between aspects of child emotional and behavioral development.

| Paternal involvement factors | | Point estimate (β)^a^ | *SE* | *P*-value |
| --- | --- | --- | --- | --- |
| Paternal parenting confidence | Paternal conflictual relationship with child | 0.501 | 0.024 | ≤.001 |
|  | Paternal enjoyment and warmth | 0.528 | 0.023 | ≤.001 |
|  |  |  |  |  |
| Paternal conflictual relationship with child | Paternal enjoyment and warmth | 0.552 | 0.019 | ≤.001 |
|  |  |  |  |  |
| Aspects of child emotional and behavioral development | | Point estimate (β)^a^ | *SE* | *P*-value |
| Emotional symptoms | Conduct problems | 0.427 | 0.031 | ≤.001 |
|  | Hyperactivity | 0.312 | 0.026 | ≤.001 |
|  | Peer problems | 0.587 | 0.028 | ≤.001 |
|  |  |  |  |  |
| Conduct problems | Hyperactivity | 0.617 | 0.024 | ≤.001 |
|  | Peer problems | 0.463 | 0.034 | ≤.001 |
|  |  |  |  |  |
| Hyperactivity | Peer problems | 0.389 | 0.028 | ≤.001 |

*Note.* ^a^ Effect size are standardised (the variance of the latent factors was fixed to ‘1’) regression coefficients (*β)*

**Table S3.** Total associations between paternal PND and child emotional symptoms, conduct problems, hyperactivity and peer problems in complete case analyses.

|  | Model estimates (*N*=4,898) | | | | | |
| --- | --- | --- | --- | --- | --- | --- |
| Effect Size ^1^ | Unadjusted model | | Adjusted^1^ | | Adjusted^2^ | |
|  | *Β* [95% CI] | *P*-value | *Β* [95% CI] | *P*-value | *Β* [95% CI] | *P*-value |
| Paternal PND (8 months) |  |  |  |  |  |  |
| Child emotional symptoms | 0.109 [0.060, 0.158] | ≤.001 | 0.079 [0.030, 0.128] | .001 | 0.032 [-0.017, 0.081] | .189 |
| Child conduct problems | 0.092 [0.039, 0.145] | .001 | 0.048 [-0.001, 0.097] | .070 | 0.012 [-0.039, 0.063] | .654 |
| Child hyperactivity | 0.104 [0.057, 0.151] | ≤.001 | 0.083 [0.038, 0.128] | ≤.001 | 0.048 [0.003, 0.093] | .035 |
| Child peer problems | 0.118 [0.063, 0.173] | ≤.001 | 0.090 [0.037, 0.143] | .001 | 0.057 [0.004, 0.110] | .034 |

*Note*. ^1^Effect size are unadjusted and adjusted regression coefficients (*B* standardized); Unadjusted models: exposure and outcome only; Adjusted^1^: Adjusted for antenatal baseline socioeconomic (paternal social class, income and type of accommodation), familial (marital status and parental conflict), paternal (age and education) characteristics and child sex; Adjusted^2^: Further adjusted for maternal PND (8 weeks).

Paternal PND: Paternal Postnatal Depression; Maternal PND: Maternal Postnatal Depression.

**Table S4.** Associations between paternal PND and paternal parenting confidence, enjoyment and warmth, and conflictual relationship with child in complete case analyses.

|  | Model estimates (*N*=4,898) | | | | | |
| --- | --- | --- | --- | --- | --- | --- |
| Effect Size ^1^ | Unadjusted model | | Adjusted^1^ | | Adjusted^2^ | |
|  | *Β* [95% CI] | *P*-value | *Β* [95% CI] | *P*-value | *Β* [95% CI] | *P*-value |
| Paternal PND (8 months) |  |  |  |  |  |  |
| Paternal enjoyment and warmth | -0.398 [-0.431, -0.357] | ≤.001 | -0.380 [-0.421, -0.339] | ≤.001 | -0.359 [-0.400, -0.318] | ≤.001 |
| Paternal conflictual relationship with child | -0.490 [-0.529, -0.451] | ≤.001 | -0.462 [-0.501, -0.423] | ≤.001 | -0.432 [-0.471, -0.393] | ≤.001 |
| Paternal parenting confidence | -0.488 [-0.531, -0.445] | ≤.001 | -0.473 [-0.516, -0.430] | ≤.001 | -0.447 [-0.492, -0.402] | ≤.001 |

*Note*. ^1^Effect size are unadjusted and adjusted regression coefficients (*B* standardized); Unadjusted models: exposure and outcome only; Adjusted^1^: Adjusted for antenatal baseline socioeconomic (paternal social class, income and type of accommodation), familial (marital status and parental conflict), paternal (age and education) characteristics and child sex; Adjusted^2^: Further adjusted for maternal PND (8 weeks).

Paternal PND: Paternal Postnatal Depression; Maternal PND: Maternal Postnatal Depression.

**Table S5.** Associations between paternal parenting confidence, enjoyment and warmth, and conflictual relationship with child and child emotional symptoms, hyperactivity and peer problems in complete case analyses.

|  | Model estimates (*N*=4,898) | | | | | |
| --- | --- | --- | --- | --- | --- | --- |
| Effect Size ^1^ | Unadjusted model | | Adjusted^1^ | | Adjusted^2^ | |
|  | *Β* [95% CI] | *P*-value | *Β* [95% CI] | *P*-value | *Β* [95% CI] | *P*-value |
| Child emotional symptoms | | | | | | |
| Paternal enjoyment and warmth | 0.097 [0.009, 0.185] | .032 | 0.077 [-0.011, 0.165] | .084 | 0.072 [-0.014, 0.158] | .102 |
| Paternal conflictual relationship with child | -0.115 [-0.209, -0.021] | .017 | -0.094 [-0.190, 0.002] | .053 | -0.062 [-0.156, 0.032] | .195 |
| Paternal parenting confidence | -0.166 [-0.274, -0.058] | .002 | -0.170 [-0.276, -0.064] | .002 | -0.156 [-0.260, -0.052] | .003 |
| Child hyperactivity | | | | | | |
| Paternal enjoyment and warmth | 0.046 [-0.034, 0.126] | .267 | 0.050 [-0.028, 0.128] | .209 | 0.051 [-0.027, 0.129] | .200 |
| Paternal conflictual relationship with child | -0.136 [-0.222, -0.050] | .002 | -0.115 [-0.201, -0.029] | .009 | -0.102 [-0.188, -0.016] | .019 |
| Paternal parenting confidence | -0.101 [-0.195, -0.007] | .035 | -0.119 [-0.209, -0.029] | .010 | -0.108 [-0.198, -0.018] | .020 |
| Child peer problems | | | | | | |
| Paternal enjoyment and warmth | 0.042 [-0.050, 0.134] | .366 | 0.035 [-0.055, 0.125] | .444 | 0.031 [-0.059, 0.121] | .497 |
| Paternal conflictual relationship with child | -0.104 [-0.202, -0.006] | .037 | -0.089 [-0.185, 0.007] | .072 | -0.069 [-0.165, 0.027] | .159 |
| Paternal parenting confidence | -0.063 [-0.179, 0.053] | .279 | -0.076 [-0.190, 0.038] | .188 | -0.063 [-0.174, 0.049] | .269 |

*Note*. ^1^Effect size are unadjusted and adjusted regression coefficients (*B* standardized); Unadjusted models: exposure and outcome only; Adjusted^1^: Adjusted for antenatal baseline socioeconomic (paternal social class, income and type of accommodation), familial (marital status and parental conflict), paternal (age and education) characteristics and child sex; Adjusted^2^: Further adjusted for maternal PND (8 weeks).

Paternal PND: Paternal Postnatal Depression; Maternal PND: Maternal Postnatal Depression.

**Table S6.** Estimates of direct and mediated effects in complete case analyses.

|  | Model estimates (*N*=4,898) | | | | | |
| --- | --- | --- | --- | --- | --- | --- |
| Effect Size ^1^ | Unadjusted model | | Adjusted^1^ | | Adjusted^2^ | |
|  | *Β* [95% CI] | *P*-value | *Β* [95% CI] | *P*-value | *Β* [95% CI] | *P*-value |
| Child emotional symptoms | | | | | | |
| 1. Total indirect effect | 0.099 [0.056, 0.142] | ≤.001 | 0.094 [0.057, 0.131] | ≤.001 | 0.071 [0.036, 0.106] | ≤.001 |
| 2. Direct effect | 0.014 [-0.064, 0.092] | .727 | -0.011 [-0.085, 0.063] | .774 | -0.035 [-0.107, 0.037] | .349 |
| 3. Total effect | 0.113 [0.054, 0.172] | ≤.001 | 0.083 [0.026, 0.140] | .004 | 0.036 [-0.021, 0.093] | .212 |
| 4. Specific indirect effects |  |  |  |  |  |  |
| Paternal enjoyment and warmth | -0.038 [-0.073, -0.003] | .033 | -0.029 [-0.062, 0.004] | .085 | -0.026 [-0.057, 0.005] | .103 |
| Paternal conflictual relationship with child | 0.056 [0.021, 0.091] | .018 | 0.043 [-0.002, 0.088] | .054 | 0.027 [-0.014, 0.068] | .197 |
| Paternal parenting confidence | 0.081 [0.028, 0.134] | .002 | 0.080 [0.029, 0.131] | .002 | 0.070 [0.023, 0.117] | .003 |
| Child hyperactivity |  |  |  |  |  |  |
| 1. Total indirect effect | 0.098 [0.063, 0.133] | ≤.001 | 0.091 [0.058, 0.124] | ≤.001 | 0.074 [0.043, 0.105] | ≤.001 |
| 2. Direct effect | 0.004 [-0.065, 0.073] | .917 | -0.010 [-0.077, 0.057] | .772 | -0.026 [-0.091, 0.039] | .433 |
| 3. Total effect | 0.102 [0.047, 0.157] | ≤.001 | 0.081 [0.030, 0.132] | .002 | 0.048 [-0.003, 0.100] | .062 |
| 4. Specific indirect effects |  |  |  |  |  |  |
| Paternal enjoyment and warmth | -0.018 [-0.049, 0.013] | .267 | -0.019 [-0.048, 0.010] | .209 | -0.018 [-0.045, 0.010] | .200 |
| Paternal conflictual relationship with child | 0.067 [0.024, 0.110] | .002 | 0.053 [0.014, 0.092] | .009 | 0.044 [0.007, 0.081] | .021 |
| Paternal parenting confidence | 0.049 [0.002, 0.096] | .037 | 0.056 [0.013, 0.099] | .011 | 0.048 [0.007, 0.089] | .022 |
| Child peer problems |  |  |  |  |  |  |
| 1. Total indirect effect | 0.065 [0.022, 0.108] | .003 | 0.063 [0.022, 0.104] | .002 | 0.047 [0.010, 0.084] | .015 |
| 2. Direct effect | 0.051 [-0.033, 0.135] | .236 | 0.032 [-0.048, 0.112] | .437 | 0.016 [-0.062, 0.094] | .685 |
| 3. Total effect | 0.116 [0.051, 0.181] | ≤.001 | 0.095 [0.034, 0.156] | .002 | 0.063 [0.002, 0.124] | .043 |
| 4. Specific indirect effects |  |  |  |  |  |  |
| Paternal enjoyment and warmth | -0.017 [-0.054, 0.020] | .367 | -0.013 [-0.048, 0.022] | .445 | -0.011 [-0.042, 0.020] | .497 |
| Paternal conflictual relationship with child | 0.051 [0.004, 0.100] | .038 | 0.041 [-0.004, 0.086] | .073 | 0.030 [-0.011, 0.071] | .160 |
| Paternal parenting confidence | 0.031 [-0.026, 0.088] | .279 | 0.036 [-0.017, 0.089] | .188 | 0.028 [-0.023, 0.079] | .268 |

*Note*. ^1^Effect size are unadjusted and adjusted regression coefficients (*B* standardized); Unadjusted models: exposure and outcome only; Adjusted^1^: Adjusted for antenatal baseline socioeconomic (paternal social class, income and type of accommodation), familial (marital status and parental conflict), paternal (age and education) characteristics and child sex; Adjusted^2^: Further adjusted for maternal PND (8 weeks).

Paternal PND: Paternal Postnatal Depression; Maternal PND: Maternal Postnatal Depression.

**Table S7.** Associations between dimensions of paternal involvement and child emotional symptoms, conduct problems, hyperactivity and peer problems, independent of other paternal involvement factors in imputed sample.

|  | Model estimates (*N*=9,628) | | | | | |
| --- | --- | --- | --- | --- | --- | --- |
| Effect Size ^1^ | Unadjusted model | | Adjusted^1^ | | Adjusted^2^ | |
|  | *Β* [95% CI] | P-value | *Β* [95% CI] | P-value | *Β* [95% CI] | P-value |
| Paternal enjoyment and warmth | | | | | | |
| Child emotional symptoms | -0.087 [-0.140, -0.034] | .001 | -0.083 [-0.136, -0.030] | .002 | -0.046 [-0.010, 0.005] | .076 |
| Child hyperactivity | -0.105 [-0.156, -0.054] | ≤.001 | -0.093 [-0.142, -0.044] | ≤.001 | -0.063 [-0.110, -0.016] | .008 |
| Child peer problems | -0.083 [-0.140, -0.026] | .005 | -0.080 [-0.137, -0.023] | .007 | -0.053 [-0.108, 0.002] | .059 |
| Paternal conflictual relationship with child | | | | | | |
| Child emotional symptoms | -0.169 [-0.222, -0.116] | ≤.001 | -0.143 [-0.120, -0.100] | ≤.001 | -0.097 [-0.148, -0.046] | ≤.001 |
| Child hyperactivity | -0.187 [-0.236, -0.138] | ≤.001 | -0.163 [-0.210, -0.116] | ≤.001 | -0.133 [-0.180, -0.086] | ≤.001 |
| Child peer problems | -0.147 [-0.206, -0.088] | ≤.001 | -0.126 [-0.183, -0.069] | ≤.001 | -0.096 [-0.153, -0.039] | .001 |
| Paternal parenting confidence | | | | | | |
| Child emotional symptoms | -0.180 [-0.239, -0.121] | ≤.001 | -0.164 [-0.221, -0.107] | ≤.001 | -0.122 [-0.179, -0.065] | ≤.001 |
| Child hyperactivity | -0.164 [-0.217, -0.111] | ≤.001 | -0.156 [-0.207, -0.105] | ≤.001 | -0.126 [-0.177, -0.075] | ≤.001 |
| Child peer problems | -0.119 [-0.184, -0.054] | ≤.001 | -0.111 [-0.174, -0.048] | .001 | -0.081 [-0.144, -0.018] | .012 |

*Note*. ^1^Effect size are unadjusted and adjusted regression coefficients (*B* standardized); Unadjusted models: exposure and outcome only; Adjusted^1^: Adjusted for antenatal baseline socioeconomic (paternal social class, income and type of accommodation), familial (marital status and parental conflict), paternal (age and education) characteristics and child sex; Adjusted^2^: Further adjusted for maternal PND (8 weeks).

Paternal PND: Paternal Postnatal Depression; Maternal PND: Maternal Postnatal Depression.
